# The case series of functional adrenal tumors: Experience of two tertiary hospitals in Yaoundé, Cameroon

**DOI:** 10.1016/j.ijscr.2020.05.097

**Published:** 2020-06-15

**Authors:** L.O. Mbouché, F.G. Epoupa Ngallé, Z. Sando, S.P. Choukem, F.F. Angwafo

**Affiliations:** a Department of Surgery, Yaoundé Gynaeco-Obstetric and Pediatric Hospital, University of Yaoundé I, Cameroon; b Department of Pathology, Yaoundé Gynaeco-Obstetric and Pediatric Hospital, University of Yaoundé I, Cameroon; c Department of Internal Medicine, Douala General Hospital, University of Dschang, Cameroon

**Keywords:** Adrenal tumor, Cushing syndrome, Adrenalectomy, Corticoid

## Abstract

**Introduction:**

Adrenal tumors are often found incidentally during abdominal imaging. Functioning adrenal tumors are less frequent than these incidentalomas discovered unexpectedly. We report treatment outcomes (major complications) of 7 cases of symptomatic adrenal masses from 2009 to 2019.

**Presentation of the cases:**

Seven cases of functioning adrenal tumors: four adenomas presenting with Cushing’s syndrome, two adrenal carcinomas, and one pheochromocytoma are described. The preoperative diagnoses were made through clinical manifestations, an increase in urinary free cortisol with normal ACTH, elevated metanephrine and enlarged masses on CT. The diagnoses were established on histopathology of adrenalectomy specimens. Adrenal insufficiency in two patients following surgery was corrected with corticoid replacement therapy. One patient died of hypovolemia the day of surgery and another from anaphylactic shock (allergy) late in the post-operative period.

**Discussion:**

Pre, intra and post-operative complications from vascular instability often complicate surgery in functioning adrenal tumors. Adrenal adenomas manifest as Cushing’s syndrome in 10–15 % of patients. They are the most common adrenal tumor although the diagnosis is most often coincidental to abdominal imaging. The incidence of adrenal adenoma increases with age, up to 7 % in the seventh decade. Laparoscopic adrenalectomy, which was not available in our hospitals then, is standard treatment for most tumors. It is alleged to have better outcomes in trained and tested hands.

**Conclusion:**

Surgery of functioning adrenal tumors demands close collaboration of multiple clinical disciplines to manage vascular instability and adrenal insufficiency, especially in resource strapped communities.

## Highlights

• This is a case series of functional adrenal tumors and treatment outcomes.
• A diversity of cases is presented with clinical, medical imaging, macroscopic and microscopic features included.
• Successful management of two cases of adrenal insufficiency following classic adrenalectomy in a low resource community is reported.

## 1 Introduction

Approximately 55 % of primary adrenal tumors are functional [1]. Depending on the type of hyperfunction, they can be classified into three major individual tumor types. Tumors with Cushing’s syndrome (those secreting glucocorticoids), tumors with primary hyperaldosteronism (those secreting mineralocorticoids), and tumors with an adrenogenital syndrome (those secreting sex steroids) [2]. Most commonly, adrenal tumors are discovered fortuitously during abdominal imaging [2] as was reported in a series of 7 incidentalomas in Cameroon in 1999 [3]. In contrast, we present a series of 7 patients with symptomatic adrenal tumors managed in the Surgery Departments of the Yaounde General Hospital and the Yaounde Gyneco-Obstetric and Pediatric Hospital over a ten-year period from 2009 to 2019. This article complies with the PROCESS guidelines for reporting surgical case series [4].

## 2 Case No 1

**Fig. 1:**
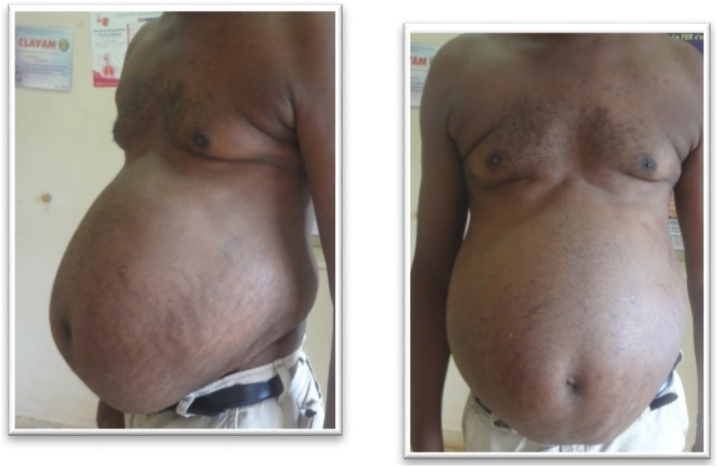
Abdominal distension with stretch marks in a 46 year old male patient (case 1).

**Fig. 2:**
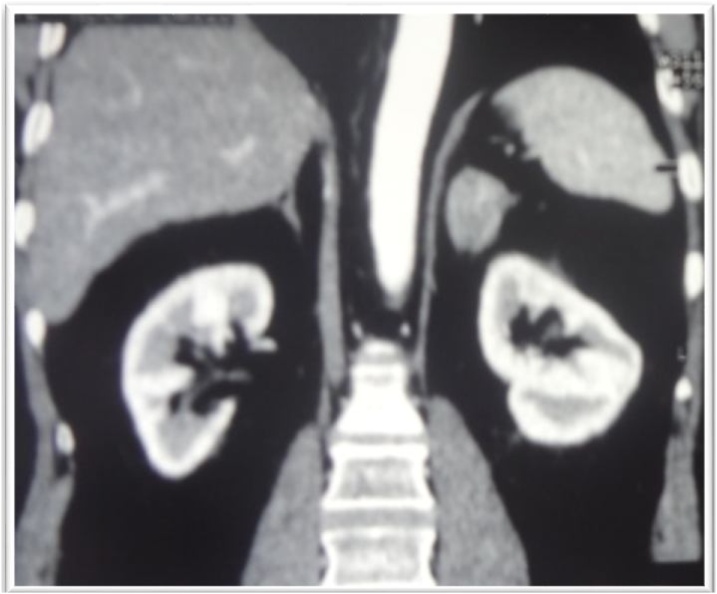
CT urogram showing left adrenal mass (case 1).

**Fig. 3:**
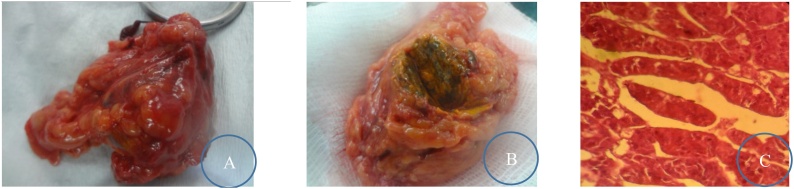
Left adrenalectomy postoperative specimen (A: en bloc, B: opened, C: under 40 objective lens; Acinar glandular trabecular architecture with giant cells having abundant granular cytoplasm) (case 1).

This is a 46-year-old male patient referred for the management of a left adrenal corticoid secreting mass. He presented with a 3 months’ duration of painless and progressing abdominal distension, discomfort, insomnia, anorexia, nocturia and rapid weight gain. He took anti-hypertensives and hypoglycemic agents for type 2 diabetes. He was a well-developed, well-nourished male with a blood pressure of 150/90 mmHg, a pulse rate of 86 beats/min, weight 90 kg, waist circumference of 112 cm, and an abdominal girth of 180 cm (Fig. 1). He had a lunate fancies, dark stretch marks on the abdomen, hyperpigmentation of the hands, feet, and trunk, and edema of the lower limbs. There was no palpable lumbar mass nor costovertebral tenderness. A CT urogram revealed a left adrenal tumor measuring 23.2 × 31.3 mm strongly enhanced with contrast (Fig. 2). Urinary free cortisol was 195 μg/24 h; ACTH <1 ng / ml; VMA: 10.61 μmol/24 h, blood glucose: 1.9 g/l. He benefited from a left subcostal adrenalectomy (Fig. 3) and postoperative corticoid supplementation. He however developed acute adrenal insufficiency due to non-compliance with opotherapy. He was stabilized in the intensive care unit and discharged 6 weeks later in good condition. The histopathology confirmed an adrenal adenoma.

## 3 Case No 2

**Fig. 4:**
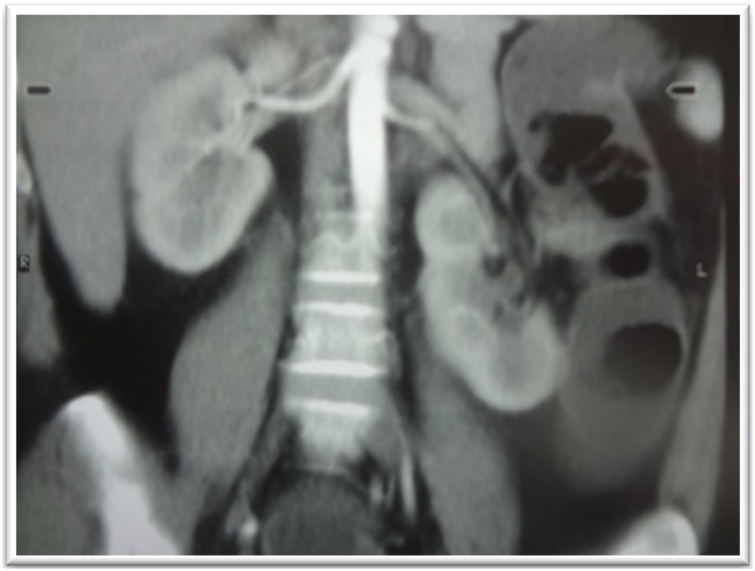
Abdominal CT urogram showing left renal malroatation and left adrenal mass (case 2).

A 37-year-old woman who presented with complaints of weight gain, galactorrhoea, hypertension for the past year and type 2 diabetes mellitus for 10 years. She was a well-developed, well-nourished with a blood pressure of 150/90 mmHg, pulse rate of 90 beats/min, lunar facies, facio-truncal obesity, abdominal stretch marks, bruises, and edema of the lower extremities. An abdominal CT scan (Fig. 4) revealed a left adrenal mass measuring 30 × 19 mm, left renal malrotation with aberrant vascularization. Free urinary cortisol was 250 μg/24 h, ACTH: 43.80 μg/mL, prolactinemia: 116 μg/mL; LH: 6.43 IU/l; FSH: 5.27 IU/l; blood glucose: 1.20 g/l.

**Fig. 5:**
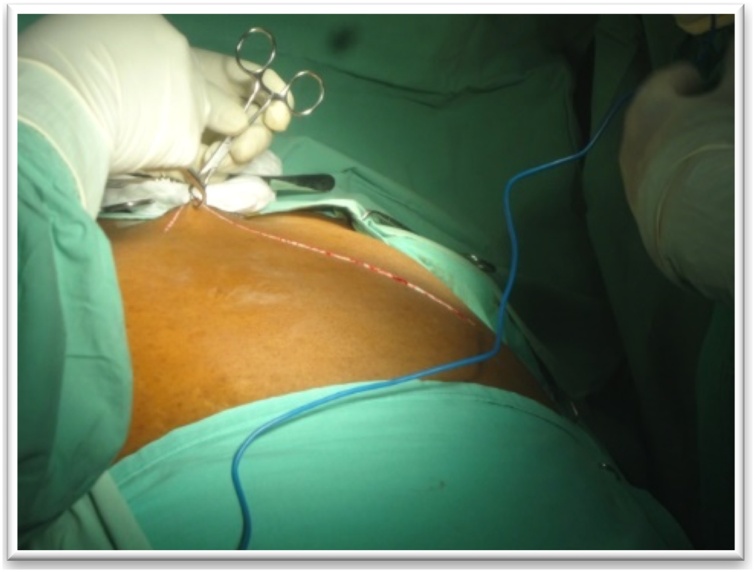
Left subcostal incision (case 2).

A left subcostal adrenalectomy (Fig. 5) was performed, and corticosteroid supplementation was initiated. Acute adrenal insufficiency developed after she arrested corticotherapy but this was rapidly corrected. The pathology report confirmed an adrenal adenoma.

## 4 Case No 3

**Fig. 6:**
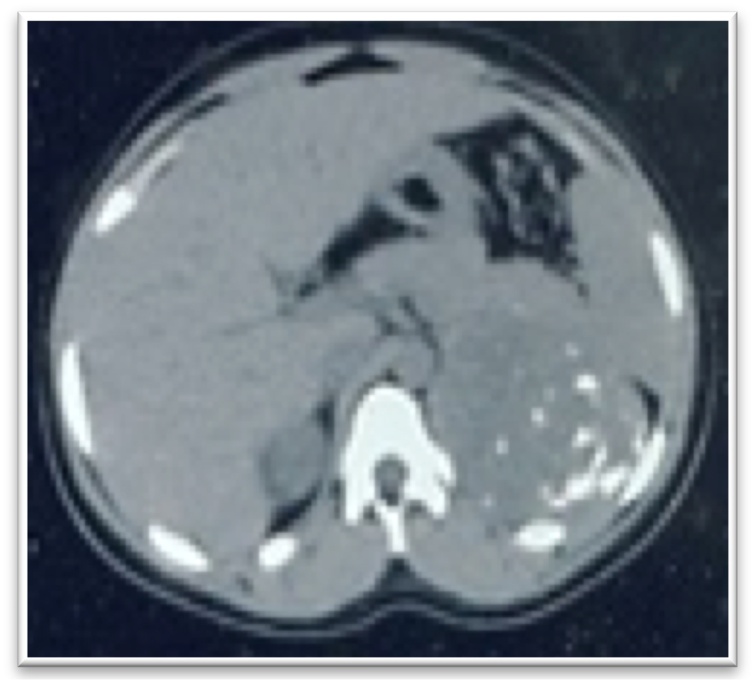
Abdominal CT showing left adrenal mass with calcifications (case 3).

A 17-year-old girl who had menarche at 12 years and menstruated for a duration of 6 months. This was followed by amenorrhoea and an unusual virilization marked by the appearance of a beard, hirsutism, a hoarse voice, marked muscular development, shoulder widening and arrest of breast development. There was a palpable left flank mass. CT scan (Fig. 6) showed a 9 × 8 cm mass with calcifications in the left adrenal region but no metastases.

There was an increase in testosterone and estradiol, with a drop in LH and FSH. Left adrenalectomy was done. The postoperative course was normal with resumption of menstruation in the first postoperative month and she gave birth to a normal child in the fourth post-operative year. The histopathology of the specimen revealed an adrenal carcinoma.

## 5 Case No 4

A 29-year-old female presented with amenorrhoea of 18 months’ duration, weight loss, progressing abdominal distension, masculinization of secondary sexual characteristics with the appearance of hirsutism, a hoarse voice and clitoral hypertrophy. A large mass occupying the right flank and hypochondrium was palpated. A hormonal profile showed elevated testosterone (6.94 ng/ml) and 17-hydroxyprogesterone (17.2 mmol / l); FSH: 5.76; LH 31, 33; oestradiol: 32.38; baseline cortisol: 157 μg/l. Abdominal CT showed a mass of the right adrenal lodge measuring 255 mm × 145 mm × 150 mm. Tumour excision was performed. The patient died from hypovolemic shock within hours of surgery.

## 6 Case No 5

**Fig. 7:**
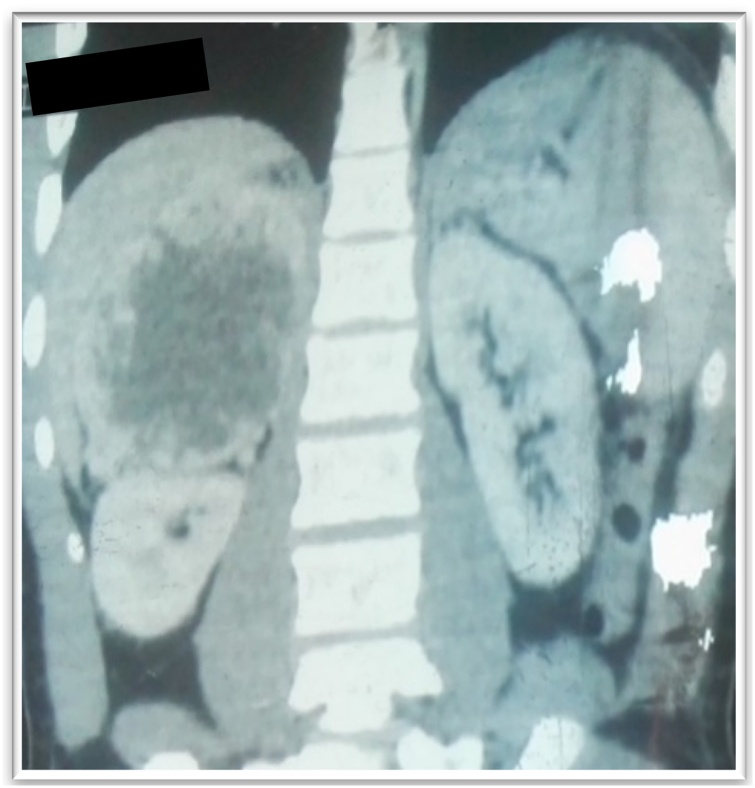
Heterogenous right adrenal mass with contrast uptake (case 5).

**Fig. 8:**
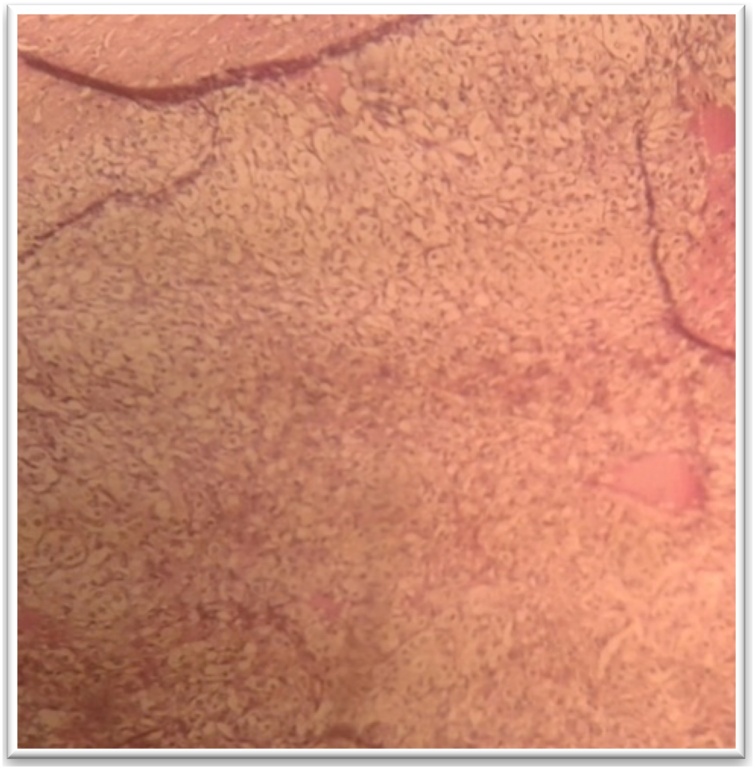
Microscopic view of Pheochromocytoma (case 5).

A 29-year-old male physician presented with progressive right flank pain for 2 years associated with pulsatile headache, palpitations, and hypersudation. On physical examination the blood pressure was 124/86 mmHg, pulse: 84 beats/min, and weight: 78 kg. The abdominal examination was normal. An abdominal CT revealed a heterogeneous right adrenal tissue mass of 115 × 83 mm (Fig. 7). Urinary free metanephrine was normal: 0.11 mmol/l; normetanephrine elevated: 42.88 mmol/l (40 times normal), elevated cortisol levels: 154 ng/mL, normal high DHEA: 48.32 mmol/l, testosterone: 8.21 ng/mL, normal blood electrolytes (Na: 138 mmol/l, K: 4.3 mmol/l; Cl: 98 mmol/l). There was severe hemodynamic instability during right adrenalectomy for pheochromocytoma confirmed on histopathological examination (Fig. 8). The patient recovered and has remained asymptomatic for the last five years.

## 7 Case No 6

**Fig. 9:**
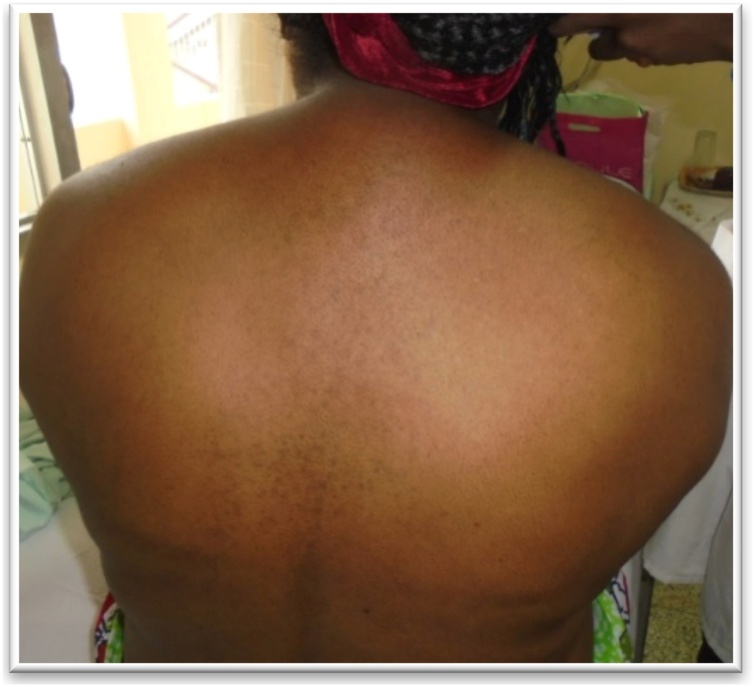
Buffalo hump (case 6).

**Fig. 10:**
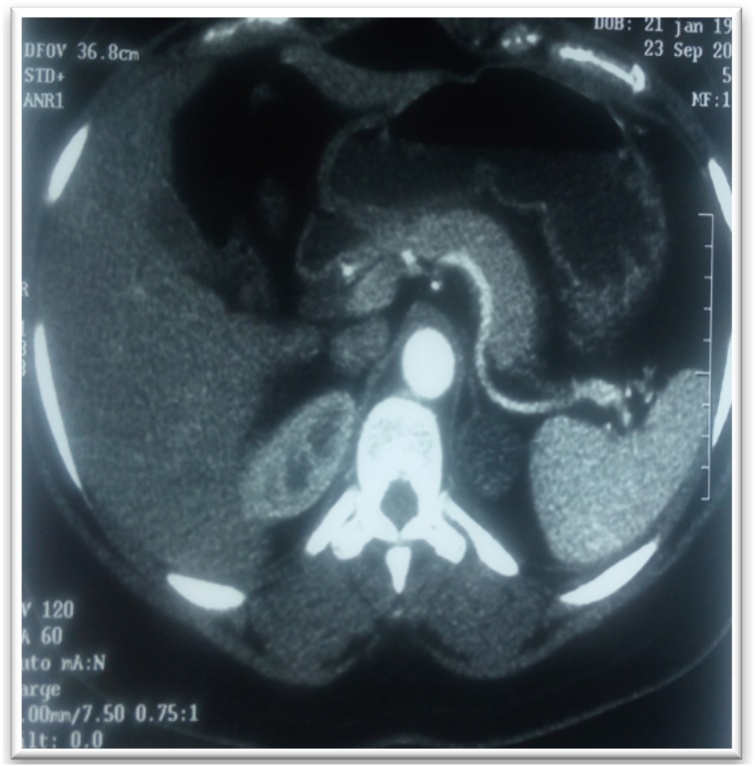
Abdominal CT showing a left adrenal mass (case 6).

**Fig. 11:**
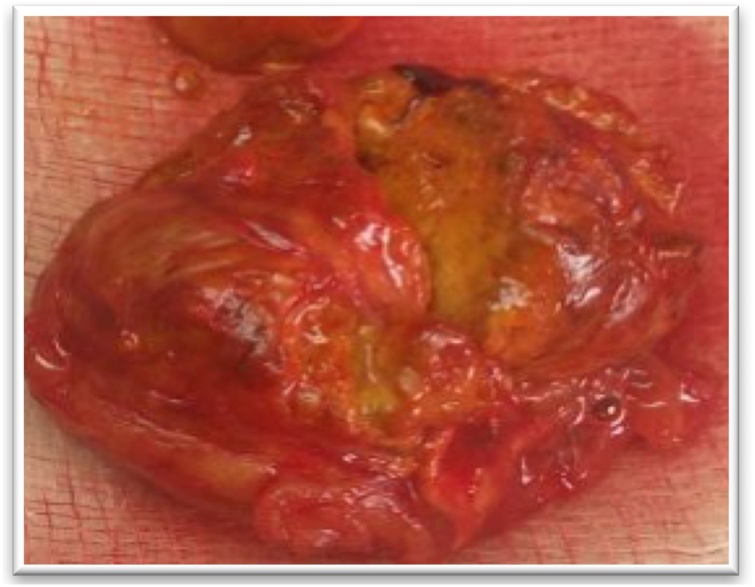
Operative sample of left adrenalectomy in patient (case 6).

**Fig. 12:**
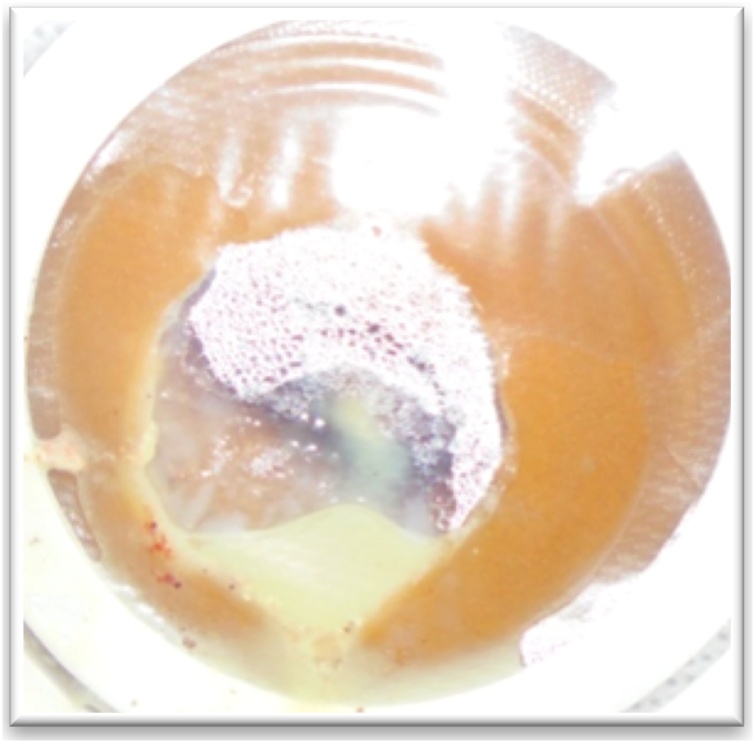
Lymphorrhoea in colostomy pouch at drainage site (case 6).

A 37-year-old woman, referred for management of an adrenal mass associated with Cushing’s syndrome. Her symptoms begun about 2 years previously with the rapid installation of bilateral pedal edema, weight gain, lightening of skin complexion, headaches, and insomnia. On physical exam, her BP was 140/100 mmHg; pulse rate 82 beats/min; weight 94 kg, and BMI 31.04 kg/m^2^. She had facio-truncal obesity, a buffalo hump (Fig. 9) and extensive stretch marks on her arms and thighs. An abdominal CT showed a left adrenal mass (Fig. 10). Free urinary cortisol was 966 nmol/24 h (4 times the normal), and ACTH was low (1.2 ng/mL). Her pre-op workup was normal and she had left adrenalectomy (Fig. 11). Post-op evolution was marked on day 10 by a low-grade lymphorrhoea (Fig. 12) through the drainage wound. The patient, however, died five weeks later from an anaphylactic reaction following a blood transfusion. Histopathology revealed an adrenal adenoma.

## 8 Case No 7

**Fig. 13:**
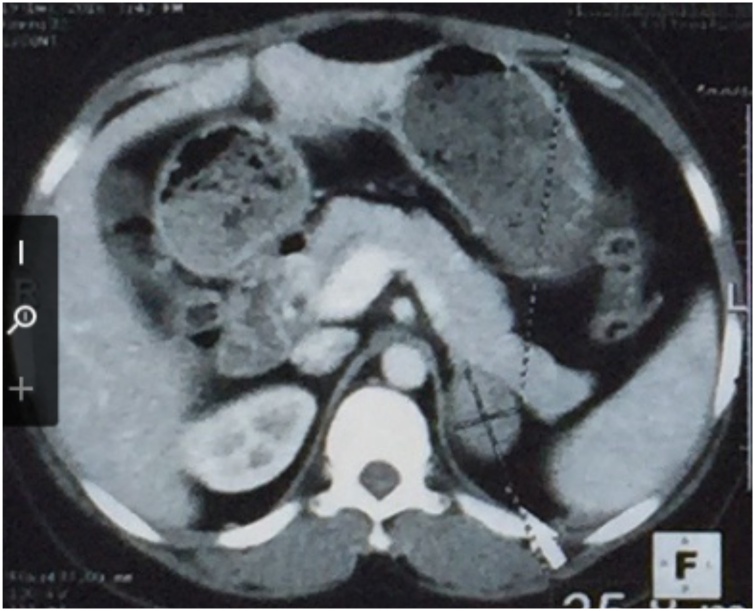
CT scan showing left adrenal mass (case 7).

**Fig. 14:**
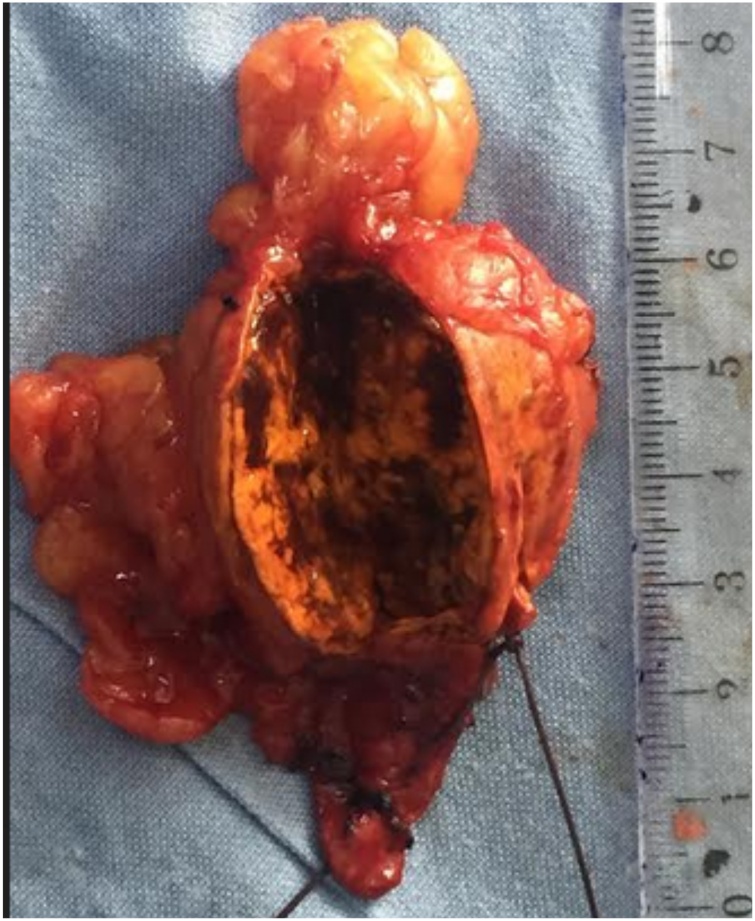
Left adrenal removed (case 7).

**Fig. 15:**
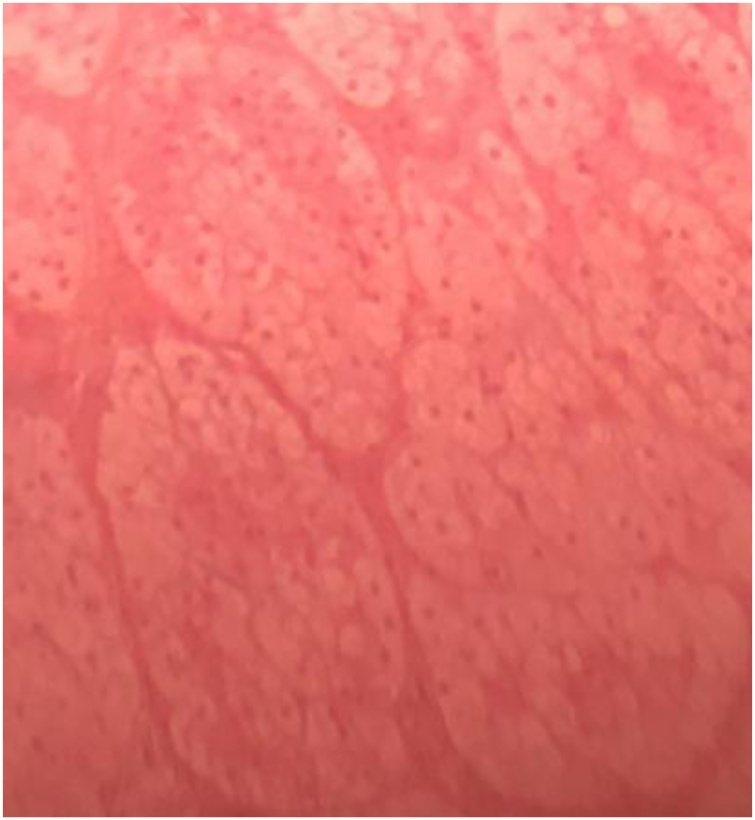
Microscopic image of adrenal adenoma: Presence of islets and small groups of epithelial cells with small non-atypical nuclei, and abundant cytoplasm (case 7).

A 25-year-old woman referred for the management of Cushing's syndrome associated with a left adrenal mass. 5 months earlier, the patient had bilateral ovarian cystectomies for polycystic ovarian disease which manifested as irregular menstrual cycle. Following surgery there was normalization of the menstrual cycle but a marked weight gain, with marked facial, truncal obesity, buffalo hump, and stretch marks all over her body.. Blood analysis revealed increased 8 a.m. serum cortisol 241 ng/mL, 4p.m serum cortisol 235 ng/mL, ACTH 1.2 ng/mL, fasting blood glucose level 0.99 g/L, serum testosterone 0.07 ng/mL, and on CT scan (Fig. 13) a 36.4 × 25cm × 34.2cm left adrenal mass. CT done for fever and persistent outflow of a brownish, viscous fluid from the adrenalectomy site confirmed abscess collection of retroperitoneal and pelvic spaces which were drained. She recovered fully and is off steroid replacement. The histopathology revealed an adrenal adenoma (Fig. 14, Fig. 15).

## 9 Discussion

Nonfunctional adrenal tumors are discovered by chance and are termed incidentalomas. We have presented seven cases of functioning adrenal tumors; four of which presented with Cushing’s syndrome, two adrenal carcinomas, and one pheochromocytoma. Adrenal adenoma manifests as Cushing’s syndrome in 10–15 % of patients [5,6]. It is the most common adrenal tumor although its diagnosis is often coincidental to abdominal imaging for another indication [2,6,7]. The incidence of adrenal adenoma increases with age, up to 7 % in the seventh decade [6]. Although the signs of Cushing's syndrome are not specific, the majority of clinical manifestations observed in our series are similar to those found in patients with Cushing's syndrome (weight gain, moon facies, truncal obesity, buffalo hump between the shoulders, pink or purple stretch marks, hirsutism, irregular or absent menstrual periods, depression, anxiety, irritability, new or worsened high blood pressure, impaired glucose tolerance). The diagnosis of Cushing’s syndrome is based on hormonal assays. The elevation of urinary free cortisol is the most reliable criterion for affirming Cushing's syndrome [2]. The sensitivity and specificity of this test varies from 96 % to 100 % [8,9]. It is recommended to carry out this assay during two or three consecutive days. More than 90 % of patients with Cushing’s syndrome have free urinary cortisol greater than 200 μg per 24 h, while normal values range from 20 to 100 μg per 24 h [2]. Only three of seven patients (42.8 %) could afford hormonal assays; this is common in resource limited communities. The ACTH assay makes it possible to verify whether hypercorticism is dependent or not on ACTH [2]. Adrenal adenomas with metabolic activity will benefit from unilateral adrenalectomy associated with perioperative and postoperative corticosteroid opotherapy while waiting for the “de-suppression” of the contralateral adrenal gland [2,6]. The two cases of acute adrenal insufficiency were subsequent to non-compliance of patients with treatment.

Adrenocortical carcinomas are rare with an incidence of 0.5–2 patients per million populations [2,6,10,11]. The tumor has a bimodal distribution with the first peak occurring in the first decade and the second peak occurring between the fourth and fifth decade [1,6]. Adrenocortical carcinoma tends to occur in patients between 30 and 70 years of age. There is an equal sex distribution but secreting tumors are more common in women (65–90%) [2,11]. Adrenal carcinomas occur either in secreting form (50–79 %) or in the non-secreting form (21–50 %). The distribution of secretory forms is thus: Cushing’s syndrome (33–54 %), mixed syndrome (Cushing + virilization) (20–24 %), isolated virilization (10–20 %), feminization (6–10 %), or hyperaldosteronism syndrome (2.5–5 %) [6]. Most carcinomas are unilateral, but bilateral occurrence is 2 %–10 % [12,13]. Non-secreting forms can manifest with abdominal pain palpable abdominal mass, weight loss, nausea, asthenia or fever [12,13]. The majority of patients present at an advanced stage, although imaging allows early detection [6]. CT is the gold standard for evaluating adrenal masses [2,6,12]. The CT scan determines the size, homogeneity, presence of calcifications (as in our 17 y. o. female), necrotic zones and metastases. The CT criteria for suspecting malignancy are large tumor size, irregular margins, and heterogeneous contrast uptake [2,12,14]. Nuclear magnetic resonance imaging is complementary to CT scan. It allows the detection of neoplastic vascular thromboses and distinction between primary carcinomas, nonfunctional adenomas, and pheochromocytomas [2]. None had an MRI because of the prohibitive cost and absence of third party insurance or universal health coverage.

The histopathological diagnosis of adrenal carcinoma is based on Weiss criteria, where at least four of the following features must be present: high nuclear grade, clear cells representing less than 25 % of the tumor, diffuse architecture (more than 33 %), necrosis, more than five mitoses in 50 fields at high magnification, atypical mitotic patterns, and any capsular, venous or sinusoid invasion. The treatment of adrenal carcinomas is essentially surgical– adrenalectomy, sometimes associated with splenectomy, nephrectomy, or thrombectomy of the inferior vena cava. Non-operable patients, are placed on iven high-dose steroidogenesis inhibitors (Mitotane) for endocrine control [15]. The prognosis for adrenal carcinoma is generally poor. The two main prognostic factors are the Tumor stage at the time of diagnosis and the quality of surgical resection [2,12]. The average survival is 18 months. The 5-year survival in localized forms in series ranges from 20 to 47 % [6,16]. The 50 % survival at 8 years is anecdotal for our series of two cases!

A case of pheochromocytoma has been described as well. Ten to 25 % of pheochromocytomas are discovered as incidentalomas [17,18]. The headache, hypersudation, and tachycardia triad are classic [17,18]. Hypertension is a frequent sign of pheochromocytomas [19]. The rich vascularity and low lipid content of pheochromocytomas help to distinguish them from adenomas by contrast enhancement of more than 10 HU on CT images [20]. The biochemical diagnosis is based on the determination of urinary catecholamines and metanephrines [21].

Laparoscopic adrenalectomy is the standard treatment for most tumors, although open surgery is recommended for large tumors and tumors with difficult access [22]. In a financial and resource-limited context, mini-invasive surgery is not always available in most health structures. All patients who received steroids after adrenalectomy were discharged and sent back to their physicians for the follow-up. However, we deplore the death of two patients: The first carried a huge expanding right painful adrenal mass for three years and died following surgery from hypovolemic shock. The second patient died 6 weeks’ post-op from anaphylactic shock in the course of a blood transfusion. Biccard and colleagues [23] have reported that surgical patients in 25 African countries are twice as likely to die when compared with the global average. This underscores the urgency of the implementation of “The G4 Alliance” programs in anaesthesia, surgery, obstetrics and trauma care [24].

## 10 Conclusion

Patients with adrenal tumors may present with sundry signs and symptoms or none. Secreting forms require precise imaging and biochemical diagnosis. Multidisciplinary management consists of adrenalectomy associated with opotherapy. Signs of acute adrenal insufficiency should be sought and managed in the immediate post-operative period. Improvements at the level of care with the introduction of minimally invasive techniques and fast tracking durable economic development within the framework of the United Nations’ “Sustainable Development Goals” would lead to earlier diagnosis and treatment to diminish the morbidity and mortality associated with functioning adrenal tumors.

## Declaration of Competing Interest

No conflict of interest.

## Funding

No funding was received for the research.

## Ethical approval

Ethical approval was obtained from the ethical committees of Yaounde gyneco-obstetric and Paediatric hospital and Yaounde General Hospital.

## Consent

Written informed consent was obtained from all the patients for publication of this case report and accompanying images. A copy of the written consent is available for review by the Editor-in-Chief of this journal on request.

We declare that no alteration was done on the images and that patient confidentiality was maintained throughout as no identifying information is contained in the article.

## Author contribution

The study was conceived by LOM and FGEN. Data collection, analysis and interpretation was done by LOM, FGEM and SPC. General supervision was done by FFA. Final manuscript was read and approved by all authors.

## Registration of research studies

1 Name of the registry: Research registry.
2 Unique identifying number or registration ID: 5600.
3 Hyperlink to your specific registration (must be publicly accessible and will be checked): https://www.researchregistry.com/browse-the-registry#home/.

## Guarantor

Fru Angwafo III, Professor of Urology at the Faculty of Medicine and Biomedical Sciences of the University of Yaoundé I, Cameroon.

## Provenance and peer review

Not commissioned, externally peer-reviewed.

## CRediT authorship contribution statement

**L.O. Mbouché:** Conceptualization, Data curation, Formal analysis, Funding acquisition, Investigation, Writing - original draft, Writing - review & editing, Methodology, Project administration, Resources, Software. **F.G. Epoupa Ngallé:** Conceptualization, Data curation, Formal analysis, Funding acquisition, Investigation. **Z. Sando:** Methodology, Project administration, Resources, Software. **S.P. Choukem:** Methodology, Project administration, Resources, Software. **F.F. Angwafo:** Supervision, Validation, Visualization, Writing - original draft, Writing - review & editing.
